# The Effect of Glucose Variability on QTc Duration and Dispersion in Patients with Type 2 Diabetes Mellitus

**DOI:** 10.12669/pjms.331.11440

**Published:** 2017

**Authors:** Yasar Sertbas, Ali Ozdemir, Meltem Sertbas, Akin Dayan, Seda Sancak, Cihangir Uyan

**Affiliations:** 1 Yasar Sertbas, MD. Doctor, Department of Internal Medicine, Fatih Sultan Mehmet Education and Research Hospital, Istanbul, Turkey; 2 Ali Ozdemir, MD. Associate Professor, Department of Internal Medicine, Fatih Sultan Mehmet Education and Research Hospital, Istanbul, Turkey; 3 Meltem Sertbas, MD. Doctor, Department of Internal Medicine, Fatih Sultan Mehmet Education and Research Hospital, Istanbul, Turkey; 4 Akin Dayan, MD, Doctor, Department of Family Medicine, Haydarpasa Numune Education and Research Hospital, Istanbul, Turkey; 5 Seda Sancak, MD. Associate Professor, Department of Endocrinology, Fatih Sultan Mehmet Education and Research Hospital, Istanbul, Turkey; 6 Cihangir Uyan, MD. Professor, Department of Cardiology, Fatih Sultan Mehmet Education and Research Hospital, Istanbul, Turkey

**Keywords:** Glucose variability, QTc dispersion, QT duration

## Abstract

**Objective:**

Glycemic variability (GV) is a new term with the episodes of hyper and hypoglycemia in diabetic patients. Both prolonged QT interval and QTd are potential risk factors for malignant ventricular arrhythmias affecting the mortality of different groups of patients including diabetes mellitus. In this study, we aimed to evaluate if the glucose variability increasing the QTc interval and QTc dispersion in type 2 diabetes mellitus.

**Methods:**

We included 275 consecutive patients with type 2 diabetes. We quantified the GV with standard deviation (SD) and coefficient of variation (CV) from 7 point glucose measures. We investigated the relationship of GV parameters with QT parameters.

**Results:**

The prevalence of prolonged QTc duration was 21%, no patients have prolonged QTc dispersion (> 80 ms). SD of the patients with prolonged QTc duration was significantly higher than the others (45.14 ±24.45 vs. 37.78 ±9.03 p<0.05). There was also a significant relationship between SD and QTc dispersion (r: 0.164; p: 0.007). There were no relationship between the QT parameters and microvascular diabetic complications. SD and HbA1c levels were significantly higher on the patients having peripheral neuropathy (p<0.005).

**Conclusion:**

The result of this study demonstratess that increased glycemic variability is associated with prolonged QTc duration and QTc dispersion. It is important to focus on targeting optimal glycemic control with GV as an additional goal point along with the traditional following parameters such as fasting-postprandial blood glucose and HbA1c.

## INTRODUCTION

Glycemic control is essential for the management of type 2 diabetes mellitus. Glycemic variability (GV) is a term in which the diabetic patients have similar mean glucose levels, although they exhibit differences in terms of both the number and degree of glucose excursions.^1^ The broad definition of GV takes into account the intraday glycemic excursions including episodes of hyper and hypoglycemia.^2^ Recently, many clinical studies have shown that glycemic variability might be related with cardiovascular diseases and mortality.^3^-^6^

The QT interval on the electrocardiogram (ECG) reflects the total duration of ventricular depolarization and repolarization. The interlead variation in duration of the QT interval on the surface ECG has been referred to as QT dispersion (QTd). Both prolonged QTc interval and QTc dispersion are potential risk factors for malignant ventricular arrhythmias affecting the mortality of different groups of patients including diabetes mellitus.^7^-^9^ There are also many studies about the influence of changes in glycemia on the length of QT parameters. Either hypoglycemia or hyperglycemia both can increase the QT duration and QTd.^10^,^11^

In this study, we aimed to evaluate the effect of glucose variability on QT interval and dispersion in type 2 diabetic patients as a predictive markers of arrhythmias and mortality.

## METHODS

The present study included 275 consecutive patients with type 2 diabetes treated in the internal medicine department of Fatih Sultan Mehmet Education and Research Hospital, Turkey between January and March 2016. The study was approved by the institutional Ethics Committee and all the participants gave written informed consent (FSM EAH-KAEK: 2015/21).

Medical documentation of all patients were systematically recorded with respect to age, sex, body weight, diabetes duration, medication and presence of diabetic microvascular complications (retinopathy, nephropathy and peripheral neuropathy).

Blood pressure was measured two consecutive times in the sitting position by an appropriate cuff size. Hypertension was diagnosed as systolic blood pressure (SBP) ≥140mmHg or diastolic blood pressure (DBP) ≥90 mmHg or the current use of antihypertensive drugs.

Diabetic retinopathy was evaluated by an opthtalmologist; diabetic nephropathy was diagnosed if patient had creatinin above 1.5mg/dl or urine protein excretion was > 300 mg/day. Peripheral neuropathy was diagnosed on the basis of neuropathy symptoms and signs of objectively abnormal results, including intensitivity to a 10g monofilament and abnormal electromyography (EMG) findings.

We quantified the glycemic variability with standard deviation (SD) and coefficient of variation (CV) from the multiple self monitoring of blood glucose (SMBG) readings taken over the course of the day with seven point glucose measures (before breakfast, two hour after breakfast, before lunch, two hour after lunch, before dinner, two hour after dinner and before sleep) CV was defined as the ratio of SD to mean glucose values expressed as a percentage.^12^ Blood glucose concentration was measured in capillary blood obtained by finger stick with a point of glucometer.

A single investigator blind to the clinical data interpreted QT intervals using the program Adobe Photoshop CS64. The QT interval was measured from the beginning of the QRS complex to the end of the downslope of the T wave (crossing the isoelectric line); when U wave present, QT interval was measured to the nadir of the curve between the T and U waves. The QT interval corrected for the previous cardiac cycle length (QTc) was calculated according to Bazett’s Formula. QTc = QT/(RR)^1/2^. QTc>440 ms was considered prolonged. QTc dispersion was calculated using the difference between the maximum and the minimum QTc in any thoracic lead. QTc dispersion >80ms was considered prolonged.^13^

Blood samples were obtained after 12 hours fasting period. Glucose was carried out by the glucose oxidase method in the auto analyzer (Architect, 16000, Abbot Diagnostic) and that of HbA1c was measured with high performance liquid chromatography (HPLC) (Trinity Biotech Premier Hb9210).

Analyses were performed using statistical package for social sciences (SPSS) version 22.0 for Windows. Data are expressed as mean (standard deviation. One-sample Kolmogorov-Smirnov test was performed to assess the distribution of data. Numerical variables in different subjects were compared by t-test or Mann-Whitney U test. Bivariate correlation analyses were made by Pearson correlation test. Probability values were two tailed, and a p-value of less than 0.05 was considered significant.

## RESULTS

There were 275 participants which included 172 female and 103 male diabetics with a mean age of 56.45±9.95. Patients’ demographic, clinical and metabolic data are presented in Table-I.

**Table-I:** Patients demographic, clinical and metabolic characteristics.

Number of patients:	275
Gender (male/female)	103/172
Age (years)	56.45 ±9.95
Diabetes duration (years)	9.76± 7.65
Body mass index (kg/m^2^)	30.80 ±5.37
Hypertension (%)	38.9
Retinopathy (%)	11.6
Nephropathy (%)	17
Peripheral neuropathy (%)	28.7
Metformin usage (%)	92.7
Sulphanylurea usage (%)	20
Insulin usage (%)	52.3
Fasting glucose (mg/dl)	165.13± 55.78
HbA1c (%)	8±1.68
SD	39.33±18.75
CV	24.03 ± 9.38
QTc	419.07± 28.54
QTc dispersion	26.43 ± 10.1

There was a significant positive correlation between SD and QTc dispersion (r: 0.164; p: 0.007). In linear regression analysis, the relation of the SD and QTc dispersion was as β= 0.088, p<0.001. (Table-II).

**Table-II:** The association between glucose variability and QT parameters.

		QTc duration	QTc dispersion
SD	r	0.116	0.164**
	P	0.055	0.007
CV	r	0.045	0.093
	P	0.457	0.124

** correlation is significant at the 0.01 level.

While the prevalence of prolonged QTc duration was 21% (QTc>440 ms), no patients have prolonged QTc dispersion (> 80 ms). We compared the clinical and metabolic characteristics of patients with prolonged QTc duration ad normals, it is shown in Table III that SD was the only parameter significantly higher on patients with prolonged QTc duration than normals (p<0.05). No other metabolic parameters were affected from the prolongation of QTc duration (Table-III).

**Table-III:** The effect of QTc prolongation on the glycemic parameters and complications.

	QTc ≤ 440 ms	QTc> 440 ms	P
SD	37.78 ± 16.63	45.14 ±24.45	0.008
CV	23.48 ± 9.03	26.09 ±10.39	0.6
Fasting glucose (mg/dl)	165.22 ±7.49	164.79 ±49.31	0.958
HbA1c (%)	7.97 ±1.71	8.23 ±1.63	0.366
Diabetes duration	9.67 ±7.01	10.09 ±9.73	0.714
Neuropathy	22.5%	6.2%	0.507
Retinopathy	9%	2.6%	0.602
Nephropathy	11.2%	2.5%	0.306

There were no relationship between the QT parameters and microvascular complications. GV or QT did not have any effect on nephropathy. Retinopathy was more prominent on patients with higher. HbA1c levels. SD and HbA1c levels were significantly higher in among patients having peripheral neuropathy (p<0.005) (Table-IV).

**Table-IV:** Relationship of GV and QT parameters with microvascular complications.

		SD	CV	HbA1c	QTc	QTc dispersion
Retinopathy (11.6%)	(-)	38.82±19.19	24.17±9.75	7.93±1.69	417.4 ±27.63	26.22 ±10.66
	(+)	42.90 ±19.82	23.21 ±8.96	8.8±1.90	425.9 ±22.89	27.56 ±10.13
	P	0.266	0.603	*0.009*	0.100	0.508
Nephropathy (13.7%)	(-)	39.10 ±19.29	24.01 ±9.53	7.96 ±1.71	419.70 ±27.99	26.21 ±10.06
	(+)	40.88 ±17.51	23.91 ±9.09	8.21 ±1.62	415.87 ±23.93	28.31 ±10.09
	P	0.592	.0.954	0.417	0.422	0.293
Peripheral Neuropathy (28.7%)	(-)	36.77 ±17.88	23.68 ±9.46	7.72 ±1.44	418.96 ±27.32	26.26 ±10.98
	(+)	44.01 ±21.13	24.54 ±9.96	8.66 ±2.09	418.34 ±27.04	26.78 ±9.70
	P	0.006	0.523	0.000	0.868	0.724

## DISCUSSION

GV with the components of the hypo and hyperglycemia attacks increase the cardiac mortality in type 2 diabetic patients. By looking the relationship of GV and the increase of QT duration and QTc dispersion, we can get information about the effect of GV on malignant ventricular arrhythmias. This study demonstrates that ventricular depolarization and repolarization are affected by glycemic variability in type 2 diabetic patients.

Robinson et al. and Marques et al. showed that experimental hypoglycemia causes an acquired long QT syndrome.^14^,^15^ Later on it was also shown that QTc interval lengthens significantly during spontaneous clinical episodes of hypoglycemia.^10^,^16^ The cause of prolonged QTc intervals and QTc dispersion during hypoglycemia is complex and multifactorial. Increased sympathoadrenal activity, high levels of cathecolamines and hypokalemia induced by hyperinsulinemia are claimed as factors to be involved.^15^,^17^,^18^

Hyperglycemia is another component of GV, has also been shown to be related with prolonged QT interval and QTc dispersion. Marfella et al. and Gordin et al. showed that acute hyperglycemia produces significant increments of QTc and QTc dispersion both in the diabetic patients and healthy individuals.^11^,^19^ Reduction of nitric oxide bioavailability and stimulation of protein kinase C were suggested as the mechanisms that cause QT prolongation in hyperglycemia.^20^-^22^

In our study the prevalence of prolonged QTc (QTc >440 ms) was 21%. On the other hand, no patients have prolonged QTc dispersion (> 80 ms). These findings were in accordance with the previous studies as the prevalence of high QTc duration and QTc dispersion ranged from 15.4 to 67% for QTc and from none to 33% for QTc dispersion.^23^-^26^ Ninkovic et al. mentioned about the GV as a risk factor of prolonged QT and QT dispersion.^27^ They used the mean amplitude of glycemic excursion (MAGE) to represent the GV. MAGE was originally developed using hourly glucose samples and it has emerged as the preferred method for assessing continuous glucose monitoring (CGM) data. We used the SD and CV as the simplest method of assessing intra-day variability of serum glucose from the multiple (7 point) SMBG readings taken over the course of the day. When we compared the glycemic parameters of the patients who have QTc parameters longer than 440 ms and below, we saw that SD was the only parameter significantly higher in the group having a longer QTc duration. Since no patient had QTc dispersion above 80 ms we looked into the correlation between the QTc dispersion and glycemic variability. SD was significantly correlated with QTc dispersion (p: 0.007; r: 0.164). Although there was no correlation between QTc duration and SD (p: 0.055), because of its proximity to the level that can be considered significant (p<0.05), we thought that the reason might be the low number of study population.

It has been suggested as the glucose variability may have been responsible for microvascular complications of diabetes. Braged et al. showed that GV was an independent predictor of peripheral neuropathy, however, no significant relationship was found between GV and the other microvascular complications (retinopathy, nephropathy).^28^ In another study painful neuropathy was found to be related to increased glucose flux.^29^ Retinopathy was also found to be related to M-FBG and HbA1c.^30^ Diabetic complications were also suggested to be higher among the patients with long QTc duration. Although some studies have suggested a higher relationship between diabetic cardiovascular autonomic neuropathy (CAN) and QT prolongation, later studies have not confirmed an association of prolonged QT and CAN.^31^,^32^ Li et al. claimed that microalbuminuria was the only diabetic microvascular complication related to QTc prolongation other than retinopathy and nephropathy.^26^ On the other hand Ninkovic et al. showed a significantly higher prevalence of retinopathy and polyneuropathy among patients with high QTc duration.^27^ In our study, we couldn’t find any relation between microvascular complications and QT parameters. While retinopathy was only found to be related with HbA1c, nephropathy hasn’t any relationship with GV parameters. SD and HbA1c were found to be significantly higher among the patients with diabetic neuropathy.

### Limitations of the study

It includes the manual assessment of the QTc interval and dispersion. Another limitation is the continuous glucose monitoring could be done instead of multiple self monitoring blood glucose. It should also be kept in mind that the superiority of available autonomic methods has not been proven for measurement of QT parameters and multiple SMBG is more feasible than CGM in daily life.^33^

In conclusion, the result of this present study suggests that increased glycemic variability associated with prolonged QTc duration and QTc dispersion, which means ventricular depolarization and repolarization are affected by glycemic variability in type 2 diabetic patients. It is important to focus on targeting optimal glycemic control with GV as an additional goal point along with the traditional following parameters such as fasting-postprandial blood glucose and HbA1c.
